# Recent Advances in Bioink Design for 3D Bioprinting of Tissues and Organs

**DOI:** 10.3389/fbioe.2017.00023

**Published:** 2017-04-05

**Authors:** Shen Ji, Murat Guvendiren

**Affiliations:** ^1^Instructive Biomaterials and Additive Manufacturing (IBAM) Laboratory, Otto H. York Department of Chemical Biological and Pharmaceutical Engineering, New Jersey Institute of Technology, Newark, NJ, USA

**Keywords:** additive manufacturing, biofabrication, tissue engineering, regenerative medicine, hydrogel, cell printing, extracellular matrix

## Abstract

There is a growing demand for alternative fabrication approaches to develop tissues and organs as conventional techniques are not capable of fabricating constructs with required structural, mechanical, and biological complexity. 3D bioprinting offers great potential to fabricate highly complex constructs with precise control of structure, mechanics, and biological matter [i.e., cells and extracellular matrix (ECM) components]. 3D bioprinting is an additive manufacturing approach that utilizes a “bioink” to fabricate devices and scaffolds in a layer-by-layer manner. 3D bioprinting allows printing of a cell suspension into a tissue construct with or without a scaffold support. The most common bioinks are cell-laden hydrogels, decellulerized ECM-based solutions, and cell suspensions. In this mini review, a brief description and comparison of the bioprinting methods, including extrusion-based, droplet-based, and laser-based bioprinting, with particular focus on bioink design requirements are presented. We also present the current state of the art in bioink design including the challenges and future directions.

## Introduction

Tissue engineering is a multidisciplinary field currently focused on two major areas: (i) developing new methods to repair, regenerate, and replace damaged tissues and organs and (ii) creating *in vitro* tissue models to better understand tissue development, disease development, and progression and to develop and screen drugs (Langer and Vacanti, 1993; Griffith and Naughton, 2002; Benam et al., 2015; Tibbitt et al., 2015; Nguyen et al., 2016; Zhang et al., 2016). Despite recent advances in tissue engineering, there is a continuous lack of tissues and organs for transplantation and a shortage for tissue models for drug discovery and testing (Bajaj et al., 2014). Conventional techniques, such as porogen-leaching, injection molding, and electrospinning, are generally recognized as the bottleneck due to limited control over scaffold architecture, composition, pore shape, size, and distribution (Murphy and Atala, 2014; Groen et al., 2016; Shafiee and Atala, 2016). 3D bioprinting enables fabrication of scaffolds, devices, and tissue models with high complexity (Murphy and Atala, 2014; Mandrycky et al., 2016; Ozbolat et al., 2016, 2017; Shafiee and Atala, 2016). 3D printing allows construction of tissues from commonly used medical images (such as X-ray, magnetic resonance imaging, and computerized tomography scan) using computer-aided design. Custom and patient-specific design, on-demand fabrication, high structural complexity, low-cost, and high-efficiency are some of the major advantages of 3D printing making it very attractive for medicine (Guillemot et al., 2010; Guvendiren et al., 2016).

3D bioprinting is a technology to fabricate constructs from living cells with or without a carrier material in a layer-by-layer manner (Dababneh and Ozbolat, 2014; Murphy and Atala, 2014; Mandrycky et al., 2016; Shafiee and Atala, 2016; Cui et al., 2017). The material that is printed is referred to as a “bioink,” which can be defined as an ink formulation that allows printing of living cells. Here, we would like to note that many of the biomaterial ink formulations are not suitable for cell printing. For instance, polycaprolactone (PCL) and poly(lactic acid) (PLA) are the most widely used biomaterials in 3D printing. However, they could only be printed at elevated temperatures in the form of a polymer melt or when dissolved in organic solvents as a polymer solution. Therefore, they are not considered as bioinks in this review, as both approaches are not suitable for live cell printing (Jose et al., 2016; Munaz et al., 2016). In this paper, we discuss the most commonly used bioinks, including cell-laden hydrogels, extracellular matrix (ECM)-based solutions, and cell suspensions (Levato et al., 2014; Adam et al., 2016; Guvendiren et al., 2016; Panwar and Tan, 2016), and give the current state of the art in bioink design with challenges and future directions. A brief description and comparison of the bioprinting methods with particular focus on bioink design requirements are also given.

## 3D Bioprinting Technologies

**Figure 1:**
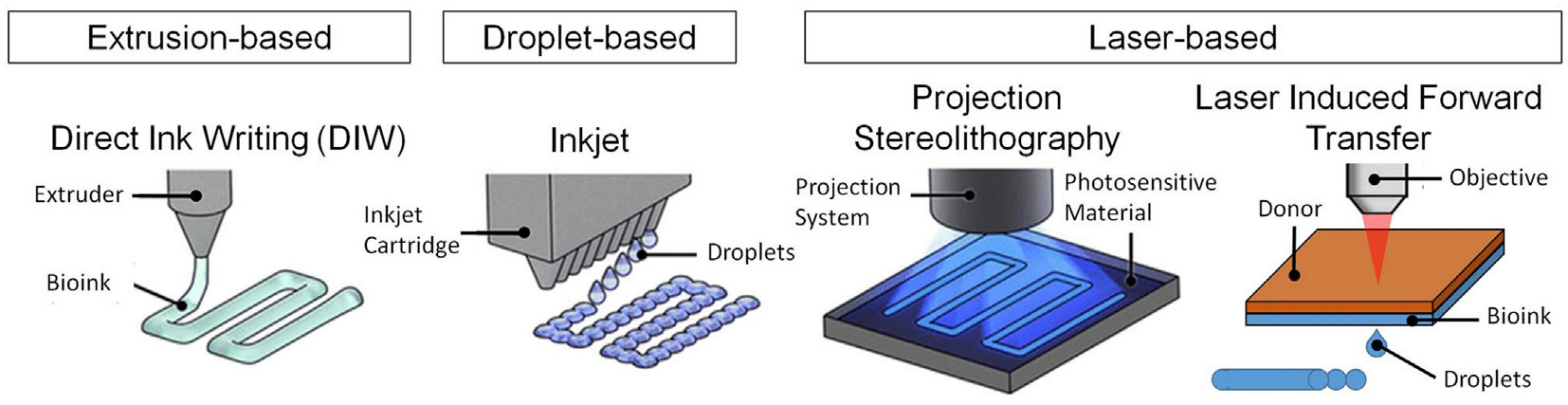
**3D bioprinting techniques for bioprinting of tissues and organs**. Figure reproduced with permission from Miller and Burdick (2016). Copyright 2016, American Chemical Society.

3D bioprinting process should be relatively mild and cell friendly as it is required to allow cell printing (Ozbolat et al., 2016, 2017). This requirement limits the number of 3D printing techniques that are suitable for bioprinting (Figure 1). It is important to note that the 3D printing technology determines the requirements for printability of a material, and not all of the 3D printing technologies are suitable for bioprinting. Currently available 3D printing technologies allow a wide range of materials to be printed using diverse ink formulations (Guvendiren et al., 2016). Fused deposition modeling (FDM) is an extrusion-based printing and utilizes synthetic thermoplastics and their composites with ceramics and metals (Turner et al., 2014). For FDM, the form of ink material is a filament, and it is extruded at elevated temperatures (140–250°C) in melt state, which eliminates FDM as an option for bioprinting. Direct ink writing (DIW) is also an extrusion-based printing and allows extrusion of high viscosity solutions, hydrogels, and colloidal suspensions (Ozbolat and Hospodiuk, 2016). DIW allows printing of cell suspensions and/or aggregates with or without a carrier. Inkjet printing is another technology for cell printing. The processing principle is deposition of polymeric solutions, colloidal suspensions, and cell suspensions, with relatively low viscosities [<10 cP (mPa⋅s)] at relatively high shear rates (10^5^–10^6^ s^−1^) in the form droplets (~50 μm in diameter) (Mironov et al., 2003; Wilson and Boland, 2003a,b; Nakamura et al., 2005; Gudapati et al., 2016). As compared to extrusion-based bioprinters, inkjet bioprinters are not readily available, yet there are commercially available inkjet print heads that are suitable for bioprinting (Nishiyama et al., 2008; Choi et al., 2011). Selective laser sintering utilizes metals, ceramics, polymers, and composites in powder form (10–150 µm in diameter) and is not suitable for bioprinting. In this technique, a directed laser beam locally melts either directly the powder or a polymeric binder onto the bed surface (Shirazi et al., 2015). Layers of fresh powder are continuously supplied after each layer is created. Stereolithography (SLA) requires a viscous photocurable polymer solution or a prepolymer, which is exposed to a directed light (such as UV or laser) to spatially cross-link the solution (Skoog et al., 2014). SLA could potentially be considered for printing live cells as long as a cell-laden prepolymer formulation is used and the photocuring takes place in a mild, cell friendly condition, which are the two major issues for SLA in bioprinting (Elomaa et al., 2015; Wang et al., 2015; Morris et al., 2017). When 3D printing technologies are considered for bioprinting, the most commonly used technologies are DIW and inkjet printing (Ozbolat et al., 2016, 2017). In addition to these technologies laser-induced forward transfer (LIFT) is also shown to be suitable for bioprinting (Barron et al., 2004a,b; Ringeisen et al., 2004; Hopp et al., 2005; Doraiswamy et al., 2006; Koch et al., 2010). In this technique, ink solution is coated onto a glass slide and coated with a laser absorption layer (metal or a metal oxide). Laser is directed to the laser absorption layer with an ablation spot size between 40 and 100 µm in diameter (Barron et al., 2004a,b; Koch et al., 2010) creating a local pressure to eject the ink layer to the substrate.

## Bioink Design

The ideal bioink formulation should satisfy certain material and biological requirements. Material properties are printability, mechanics, degradation, and functionalizability. Biological requirements mainly include biocompatibility, cytocompatilibilty, and bioactivity. When material properties are considered, printability is the most important parameter. Printability comprises two parts: (i) the processability of the bioink formulation and (ii) the print fidelity associated with the mechanical strength of the printed construct to self-sustain a 3D structure post-printing. Depending on the printing process, printability could potentially involve solution viscosity, surface tension, and cross-linking properties. Viscosity is a crucial parameter for a bioink formulation as it affects both the print fidelity and cell encapsulation efficiency. High viscosity polymer solutions are less likely to flow easily so that the printed structure could hold its shape at longer times post-printing. However, they require higher pressures to flow, limiting the gage size and smallest achievable print size (mainly for DIW). In this regard, Tirella et al. (2009) investigated the processing window for alginate hydrogels using pressure-assisted microfabrication (DIW technique). They successfully developed a 3D phase diagram showing the interplay between bioink viscosity, print velocity, and applied pressure to obtain high print fidelity (Tirella et al., 2009). The bioink formulation is preferred to have a tunable viscosity to be compatible with different bioprinters. For instance, bioinks for inkjet or droplet-based bioprinters have viscosity values close to 10 mPa⋅s (Gudapati et al., 2016); the viscosity of bioinks for extrusion-based DIW bioprinting ranges from 30 to 6 × 10^7^ mPa⋅s (Hölzl et al., 2016; Ozbolat et al., 2016, 2017); for laser-assisted bioprinting, the bioink viscosity is in the range of 1–300 mPa⋅s (Guillotin et al., 2010; Hölzl et al., 2016). For high viscosity bioinks used in extrusion and droplet-based print, the shear-thinning characteristic is desired to compensate for the high shear stress associated with high viscosity. The overall mechanics, i.e., achievable stiffness, is important not only to create self-supporting constructs but also to control and direct cellular behavior. Degradation is important for the functional integration of the printed construct *in vivo* by enabling cells to gradually replace the construct with their ECM. Both the bioink and the degradation products should not contain materials that induce inflammatory host response when implanted. Functionalizability is required to incorporate biochemical cues, i.e., bioactivity, to direct cellular behavior, such as adhesion, migration, and differentiation. In addition to biocompatibility and cytocompatibility, high cell viability, both prior- and post-printing, is crucial for the ink formulation. In addition to bioink design, a recent study showed the importance of the print substrate for live cell inkjet printing. In this work, computational and experimental studies confirmed that the stiffness of the print substrate directly influences the impact forces acting on the droplet, which affects the overall cell survival (Tirella et al., 2011). Below we will discuss the commonly used bioinks including current state of the art in ink design.

## Currently Available Bioinks

**Figure 2:**
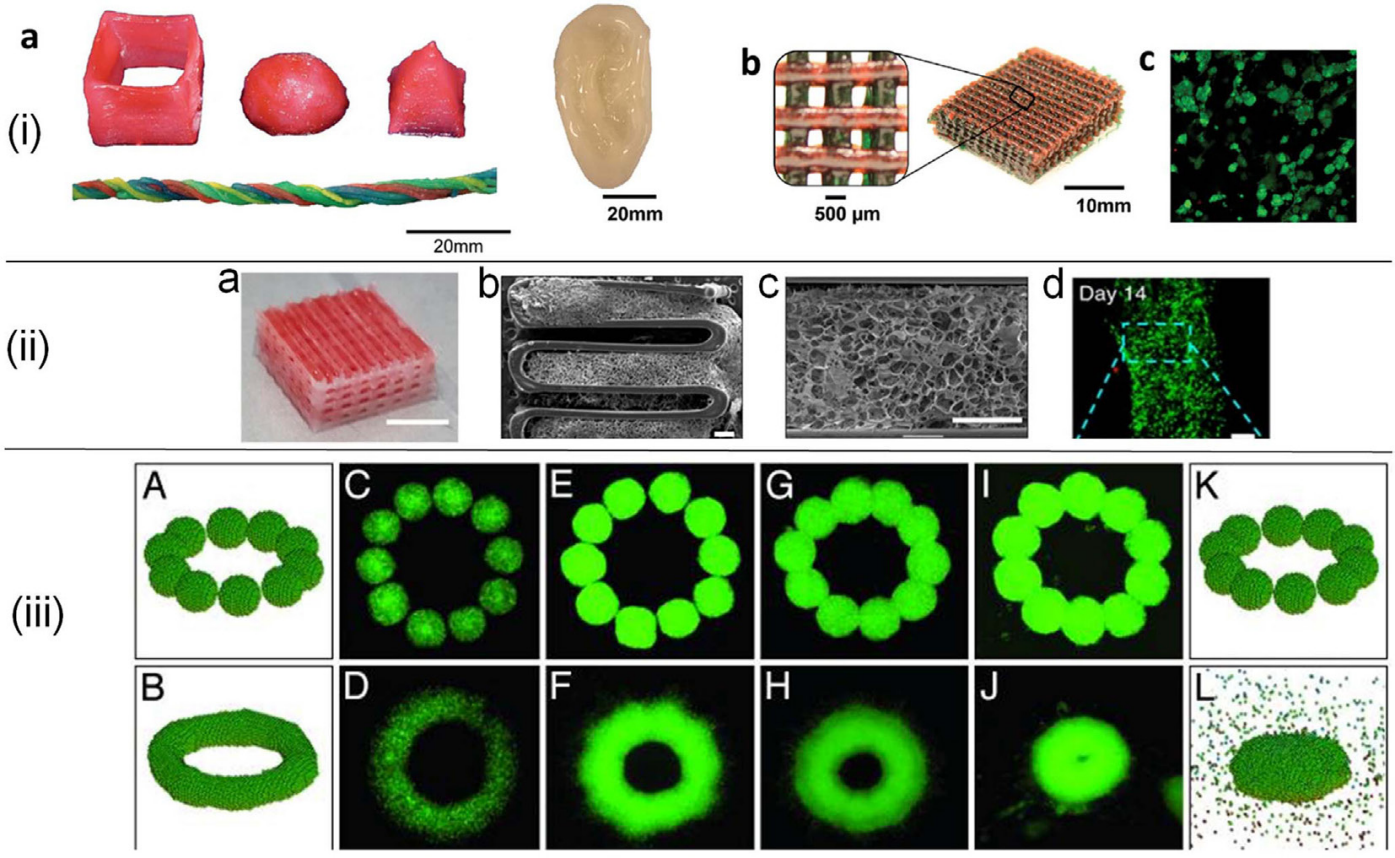
**(i)** 3D printed constructs in various forms (a,b) using poly(ethylene glycol)–alginate–nanoclay hydrogels. Red food dye was incorporated into some of the bioink formulations for visibility. Live/dead assay of cells (c) in a collagen infused mesh from (b). Reprinted with permission from Hong et al. (2015). Copyright 2015, John Wiley and Sons. **(ii)** Tissue construct printed from decellularized extracellular matrix (dECM) (a), SEM images of hybrid constructs from dECM supported with polycaprolactone framework (b,c), and fluorescent images of cells (d). Scale bars are 5 mm for (a), 400 µm for (b,c), and 100 µm for (d). Adapted with permission from Pati et al. (2014). Copyright 2014, Nature Publishing Group. **(iii)** Cell aggregate (500-µm average diameter) configurations in simulations (A,B,K,L) and experiments. C–J correspond to cell aggregates embedded in a neurogel with RGD fragments (C,D) and collagen gels of concentration 1.0 mg/ml (E,F), 1.2 mg/ml (G,H), and 1.7 mg/ml (I,J). Figure adapted with permission from Jakab et al. (2004). Copyright 2004, National Academy of Sciences.

The most commonly used bioinks for tissue and organ printing are cell-laden hydrogels, decellularized extracellular matrix (dECM)-based solutions, and cell suspensions (Figure 2). Cell-laden hydrogels are particularly attractive due to their tunable properties and their ability to recapitulate the cellular microenvironment (Fedorovich et al., 2007). ECM-based bioink formulations or decellulerized tissue inks are an emerging field due to their inherent bioactivity and ease of formulation into a printable bioink (Pati et al., 2014). Cell suspension inks based on cell aggregates are a viable option to create scaffold-free biological constructs (Forgacs and Foty, 2004; Marga et al., 2007).

### Cell-Laden Hydrogels

Cell-laden hydrogels are the most commonly used bioinks as they can be easily formulated for extrusion-based (DIW), droplet-based (inkjet), and laser-based (SLA and LIFT) bioprinting technologies. Cell-laden hydrogel bioink formulations utilize natural hydrogels such as agarose, alginate, chitosan, collagen, gelatin, fibrin, and hyaluronic acid (HA), as well as synthetic hydrogels such as pluronic (poloxamer) and poly(ethylene glycol) (PEG), or blends of both. Natural hydrogels offer inherent bioactivity except for agarose and alginate and display a structural resemblance to ECM. For instance, fibrin and collagen hydrogels with inherent filamentous structure display strain-stiffening property, mimicking the non-linear elastic behavior of the soft tissues in our body (Gardel et al., 2004; Storm et al., 2005). Synthetic hydrogels permit but do not promote cellular function, yet there are many ways to tether bioactive cues into synthetic hydrogels (Guvendiren and Burdick, 2013). When compared to natural hydrogels, synthetic hydrogels generally offer tunable mechanical properties. Many natural polymers (such as gelatin and HA) have functionalizable backbone side chains enabling them to be functionalized with chemical moieties to induce cross-linking (chemical- and/or photo-cross-linking) or additional bioactivity (Burdick and Prestwich, 2011). Blends of synthetic and natural polymers have been used to develop mechanically tunable hydrogels with user-defined bioactivity. Finally, the mechanical properties and/or bioactivity can also be tuned by incorporating small amounts of nanoparticles into bioink formulation (Ribeiro et al., 2015).

Usually, all hydrogel bioink formulations require printing of a polymer solution followed by subsequent cross-linking. This requires a highly viscous polymer solution (polymer wt% >3%) and rapid cross-linking to develop self-supporting structures. There are two forms of cross-linking: physical and chemical cross-linking. Physical cross-linking is a non-chemical approach that utilizes hydrophobic interactions, ionic interactions, and hydrogen bonding. Chemical cross-linking relies on the formation of covalent bonds, which could be a radical polymerization (such as photo-cross-linking) or Michael-type addition reaction. The chemically cross-linked hydrogels form a mechanically robust network as compared to the physically cross-linked hydrogels, which is particularly important for the stem cell behavior including differentiation (Huebsch et al., 2010; Khetan et al., 2013).

Pluronic and PEG are the most common synthetic polymers for bioprinting. Pluronic, a poloxamer-based triblock copolymer composed of two hydrophobic groups between a water-soluble group, has been widely used in extrusion-based bioprinting as it gels at room temperature but flows at temperatures below 10°C. However, it is not very stable and erodes within hours. Thus, it is generally used as a supporting material (Kang et al., 2016). Lewis Lab took an advantage of this property and printed pluronic within a photopolymerizable hydrogel to create micro channels (Wu et al., 2011). Müller et al. (2015) developed an acrylated pluronic to create UV cross-linked stable gels post-printing. The most common forms of PEG for bioinks are PEG-diacrylate (PEG-DA) and PEG-methacrylate, which are suitable for extrusion-based, droplet-based, and laser-based printing technologies (Cui et al., 2012; Hribar et al., 2014; Wüst et al., 2015). PEG is hydrophilic and not adhesive to proteins and cells; therefore, it requires blending with other natural polymers or functionalization with biochemical cues. It is possible to form strong robust hydrogels using PEG-based polymers. For instance, Hockaday et al. (2012) printed aortic valve geometries using PEG-DA hydrogels blended with alginate and achieved 10-fold range in elastic modulus from ~5 to ~75 kPa. Hong et al. (2015) reported 3D printing of tough and biocompatible, cell-laden PEG–alginate–nanoclay hydrogels infused with collagen. Rutz et al. (2015) developed partially cross-linked PEG-based multi-material bioink formulations with tunable viscosity to enhance print fidelity and secondary cross-linking ability to stabilize the constructs.

Alginate is one of the most commonly used natural polymers to formulate bioinks for inkjet and DIW printing. For inkjet printing, calcium chloride is jetted onto alginic acid solution (Boland et al., 2007). For extrusion-based printing, alginate is printed as a viscous solution, and the constructs are exposed to CaCl_2_ solution to induce post-printing cross-linking. Alginate is not cell adhesive, thus it is generally blended with other natural polymers (e.g., gelatin and fibrinogen) to induce cell adhesion and biological activity (Xu et al., 2009; Jia et al., 2014; Yu et al., 2014; Lim et al., 2016; Pan et al., 2016). Note that, the majority of the natural polymers are used as a component of bioink formulation. HA and gelatin that have been utilized extensively in the form of functionalized polymers thus fall into the synthetic polymer category, which is discussed below.

Gelatin is commonly used in the form of gelatin methacryloyl (GelMA)-based hydrogel for DIW (Bertassoni et al., 2014; Loessner et al., 2016). Lim et al. (2016) recently reported a visible light photo-cross-linking system to minimize the oxygen inhibition in photopolymerized GelMA hydrogels. They reported higher print fidelity and cell viability for ruthenium/sodium persulfate visible photo-initiator as compared to UV photo-initiator Igracure 2959. Similar to gelatin, HA has been modified in many ways to create cell-laden bioinks (Highley et al., 2015; Rodell et al., 2015; Ouyang et al., 2016). For instance, Burdick lab reported HA-based supramolecular hydrogels cross-linked by cyclodextrin–adamantane host–guest interactions, which are capable of shear-thinning and self-healing (Highley et al., 2015). The non-covalent bonds allow direct writing of inks into support gels. HA hydrogels were developed to display both shear-thinning behavior due to guest–host bonding and stabilization post-printing *via* UV-induced covalent cross-linking (Ouyang et al., 2016). Supramolecular hydrogels are particularly attractive for extrusion-based printing as they could flow under shear and self-heal immediately after printing, leading to high print fidelity. In addition to guest–host bonding, self-assembling peptides (Raphael et al., 2017) and polypeptide–DNA hydrogels (Li et al., 2015) are other emerging candidates for bioink design.

### Cell Suspension Bioinks

Modified inkjet printers have long been used to print cells into cellular assemblies. For instance, endothelial cells were printed from cell suspension (1 × 10^5^ cells/ml) in growth media (Wilson and Boland, 2003a,b). Bioprinting of scaffold-free constructs utilizes cell aggregates in the form of mono- or multicellular spheroids as a bioink (Mironov et al., 2003; Norotte et al., 2009; Jakab et al., 2010; Christensen et al., 2015). The bioink formulation undergoes a fully biological self-assembly without or in the presence of a temporary support layer (Norotte et al., 2009). This technique relies on tissue liquidity and fusion, which allow cells to self-assemble and fuse due to cell–cell interactions (Forgacs et al., 1998; Jakab et al., 2004; Fleming et al., 2010). For instance, Norotte et al. developed spheroids and cylinders of multicellular aggregates with controlled diameter in the range of 300–500 µm and showed that post-printing fusion led to single- and double-layered vascular tubes. Organovo is the first medical research company that uses a similar approach to create functional human tissues toward *in vitro* disease models. The company has developed liver models using high density bioinks from parenchymal cells or non-parenchymal cells that are printed *via* extrusion-based printing (Nguyen et al., 2016). Tissues were allowed to mature in a bioreactor for at least 3 days to form scaffold-free tissues. Levato et al. (2014) developed an alternative approach by combining bioprinting with microcarrier technology, which allowed extensive expansion of cells on cell-laden PLA-based microcarriers. Tan et al. (2016) used poly(d,l-lactic-co-glycolic acid) porous microspheres enabling cells to adhere and proliferate before printing.

### dECM-Based Bioinks

Decellularized extracellular matrix-based bioinks involve decellularization of a tissue of interest by removing the cells while preserving the ECM. The ECM is then crushed into a powder form and dissolved in a cell friendly buffer solution to formulate the bioink. A carrier polymer could be used to increase the solubility, to tune the viscosity, or to induce/enhance post-cross-linking of the bioink. In this regard, Pati et al. (2014) printed 3D constructs using dECM-based bioinks supported by a PCL framework. For this purpose, dECM was obtained from fat, cartilage, and heart, using a combination of physical, enzymatic, and chemical processes. These ink materials were initially solubilized in an acidic buffer, and pH was adjusted to accommodate cells. This formulation was soluble at 10°C and gelled at 37°C. Following this study, the same group showed that the dECM bioink can be pre-gelled using vitamin B2-induced covalent cross-linking (Jang et al., 2016a,b,c). Using this approach, a 3D printed cardiac patch composed of multiple-cell lines including human cardiac progenitor cells and mesenchymal stem cells was developed (Jang et al., 2016a,b,c). Although dECM bioinks provide novel opportunities to fabricate tissue specific constructs, the decellularization process requires multiple steps including precise quantification of the DNA and the ECM components, making it a costly approach.

## Summary and Future Perspectives

3D printing has a strong potential to become a common fabrication technique in medicine as it enables fabrication of modular and patient-specific scaffolds and devices, and tissue models, with high structural complexity and design flexibility (Murphy and Atala, 2014; Jang et al., 2016a,b,c; Kang et al., 2016; Kuo et al., 2016; Zhang et al., 2016). There is a significant interest in designing novel bioink formulations toward the goal of achieving the “ideal” bioink for each bioprinting technology (Hölzl et al., 2016). Cell-laden hydrogels are the most common bioinks, offering novel strategies including multi-material printing, shear-thinning capability, and sequential cross-linking toward self-supporting constructs. dECM-based bioinks provide an alternative approach utilizing decellulerized tissues, yet the processing of decellulerized tissue increases the cost of the bioinks. Cell aggregate printing enables direct printing of cells into tissue constructs, but the size of these constructs is currently limited as the process requires large quantities of cells. In addition to bioink development, there is also need for bioprinters with high resolution, which is particularly important to develop vascularized constructs. Considering future perspectives, supramolecular hydrogels with reversible cross-linking mechanism (Rodell et al., 2015) and stimuli responsive materials for biomimetic 4D printing (Sydney Gladman et al., 2016) are potentially the most interesting candidates for bioink design. Finally, there are still many regulatory challenges to move the 3D bioprinted constructs into clinic.

## Author Contributions

SJ and MG wrote the manuscript, and MG edited the manuscript.

## Conflict of Interest Statement

The authors declare that the research was conducted in the absence of any commercial or financial relationships that could be construed as a potential conflict of interest. The reviewer, AA, and handling Editor declared their shared affiliation, and the handling Editor states that the process nevertheless met the standards of a fair and objective review.
